# Reverse lectin ELISA for detecting fucosylated forms of α1-acid glycoprotein associated with hepatocellular carcinoma

**DOI:** 10.1371/journal.pone.0173897

**Published:** 2017-03-15

**Authors:** Eva Åström, Per Stål, Robin Zenlander, Pia Edenvik, Catharina Alexandersson, Mats Haglund, Ingvar Rydén, Peter Påhlsson

**Affiliations:** 1 Department of Clinical and Experimental Medicine, Linköping University, Linköping, Sweden; 2 Department of Medicine, Division of Gastroenterology and Hepatology, Karolinska Institutet, Karolinska University Hospital, Stockholm, Sweden; 3 Department of Infectious Diseases, Kalmar County Hospital, Kalmar, Sweden; 4 Department of Clinical Chemistry, Kalmar County Hospital, Kalmar, Sweden; Drexel University College of Medicine, UNITED STATES

## Abstract

Altered fucosylation of glycoproteins is associated with development of hepatocellular carcinoma (HCC). Lectins have been commonly used to assay changes in fucosylation of plasma glycoproteins. In the present study a recombinantly engineered form of the fucose binding lectin *Aleuria aurantia* (AAL) consisting of a single binding site for fucose (S2), was used to construct a reverse lectin ELISA method. Microtiter plates coated with the S2 lectin were used to capture glycoproteins from plasma samples followed by antibody detection of S2-bound fucosylated α1-acid glycoprotein (S2-bound AGP). The method was used to compare the level of S2-bound AGP in serum samples from a small cohort of patients with hepatitis, cirrhosis or HCC. Using the reverse S2 lectin ELISA it was shown that the levels of S2-bound AGP was significantly higher in HCC patients compared to non-cancer patients and that there was also a significant elevation of S2-bound AGP in HCC patients compared to cirrhosis patients. There was no correlation between the level of S2-bound AGP and total AGP concentration. The performance of S2-bound AGP in differentiating HCC from cirrhosis samples or hepatitis samples were compared to other markers. A combination of S2-bound AGP, α-fetoprotein and AGP concentration showed performances giving area under receiver operating curves of 0.87 and 0.95 respectively.

## Introduction

Primary liver cancer (hepatocellular carcinoma, HCC) is one of the most prevalent human cancers and the third deadliest. Most cases of HCC develop on the background of liver cirrhosis and the major etiology for developing cirrhosis and HCC is chronic hepatitis B virus (HBV) or hepatitis C virus (HCV) infection [1–3].

The most commonly used serum biomarker to diagnose and measure progression of HCC is α-fetoprotein (AFP). However the diagnostic power of AFP to detect HCC is limited. Another biomarker, des-γ-carboxy prothrombin (DCP) has shown somewhat better sensitivity than AFP in some clinical studies [4]. However, both AFP and DCP show insufficient sensitivity and specificity to be used as screening markers and their use as diagnostic markers in surveillance is not recommended in international guidelines [5]. Ultrasound has better sensitivity and specificity (60–80%) but suffers from high costs and inability to detect tumors at an early stage [6]. Thus there is a need to develop more sensitive and specific diagnostic markers to detect HCC.

Glycosylation changes such as increases in fucosylation of plasma proteins is a common finding associated with both cirrhosis and HCC development. The most well studied example is the increase in a core-fucosylated form of AFP, AFP-L3, which has been shown higher specificity for HCC than using AFP alone [7].

Mass spectrometry analyses have revealed specific differences in plasma protein fucosylation between liver cirrhosis and HCC patients [8–13]. Specifically, it has been shown that whereas there is an increase in plasma protein fucosylation in both cirrhosis and HCC patients, HCC patients express plasma proteins with more multifucosylated structures. Lectins, such as the fucose-binding lectin AAL, have been used to study these changes. However, differentiation between patients with HCC and patients with liver cirrhosis is often poor [14, 15].

AAL is composed of two identical subunits, where each subunit contains five binding sites for fucose [16]. AAL displays a broad specificity for fucosylated oligosaccharides and binds to oligosaccharides with fucose linked α1–6, α1–2, α1–3 and α1–4, including sialylated and fucosylated structures such as SLe^x^ and SLe^a^. The five different binding sites differ in binding specificity and affinity. Therefore the multivalent nature of AAL may reduce the accuracy and performance of an analytical assay for measuring specific glycoforms.

To overcome some of these problems we have previously produced a recombinant form of AAL comprising only binding site 2, (S2) [17]. This monovalent lectin showed a more restricted binding towards fucosylated oligosaccharides with reduced binding towards sialylated/fucosylated oligosaccharides compared to AAL. It also showed a general lower affinity towards fucosylated structures, with highest affinity towards multifucosylated oligosaccharides and oligosaccharides containing α1–6 linked fucose.

In the present study, we used immunoprecipitation to compare binding of the plasma protein α1-acid glycoprotein (AGP), a highly glycosylated plasma glycoprotein carrying five complex-type N-glycans, from patients with different liver diseases to AAL and S2. We found that whereas AAL bound to AGP in all samples, S2 was more restricted in its binding and primarily bound to AGP in samples from HCC-patients.

Based on this finding we developed a reverse lectin ELISA for assaying AGP with HCC-specific glycosylation.

## Material and methods

### Patient samples

Plasma samples from 32 patients with liver cirrhosis (LC) and 28 patients with HCC were collected at the Karolinska University Hospital at Huddinge and plasma samples from 32 patients with chronic hepatitis were collected at the Infectious Disease Clinic at Kalmar County Hospital (Table 1). All included HCC samples were collected before treatment (Sorafenib treatment < 1 month). Five of the HCC samples were from patients with recurrent HCC. None of the patients with liver cirrhosis had developed any signs of HCC 6 months after sampling. The patients with hepatitis were determined non-cirrhotic. A normal sample consisting of a pool of plasma from blood donors with no signs of liver disease was used as a control. Written consent was obtained from all patients and the study was approved by ethical committees at Linköping University and the Karolinska Institute.

**Table 1:** Clinical features of patients used in the study.

Disease diagnosis^a^	HCC^b^	Cirrhosis^b^	Hepatitis^c^
Number	28	32	32
^d^Etiology % (HBV/HCV/ALC/NAFLD/Other)	4/50/18/21/7	0/28/16/19/37	59/41/0/0/0
Gender % (M/F)	93/7	81/19	47/53
Age (Mean)	66.5 ± 10.1	62.1 ± 12.2	44.9 ± 9.8
^e^AFP level (mean kU/L)	5582 ± 19068	4 ± 3	6 ± 19
^f^AGP level (mean mg/ml)	1.0 ± 0.7	0.7 ± 0.3	0.7 ± 0.2
^g^BCLC stage % (0/A/B/C/D)	7/32/11/46/4	^h^N/A	N/A
Child Pugh % (A/B/C)	61/36/4	47/34/19	N/A

^a^Samples were provided coded from Karolinska University Hospital and the Infectious Disease Clinic at Kalmar County Hospital

^b^HCC diagnosis was determined according to the guidelines of EASL and AASLD for a cirrhotic liver. For non-cirrhotic livers HCC diagnosis was determined by liver biopsy. Cirrhosis was determined with Fibroscan (>Z14kPa) and ultrasound.

^c^Patients classified as HBV or HCV were defined as those with HBsAg positivity or HCV-RNA positivity but no evidence of cirrhosis

^d^Etiology: HBV, hepatitis B virus; HCV, hepatitis C virus; ALC, alcohol induced liver disease; NAFLD, non-alcoholic liver disease, including NASH; Other, autoimmune hepatitis, cryptogenic liver disease, hemochromatosis, primary biliary cirrhosis and healthy livers

^e^AFP was determined using standard methods (COBAS, Roche Diagnostics)

^f^AGP concentrations were determined using standard methods (ProSpec, Siemens)

^g^HCC staging was determined using the Barcelona-Clinic Liver Cancer (BCLC) classification

^h^N/A, not available

### Production of polyclonal anti-human-α1 acid glycoprotein 058

A polyclonal anti-human α1-acid glycoprotein antibody 058 was produced at Agrisera antibodies (Vännäs, Sweden). Shortly, a synthetic peptide corresponding to the C-terminal amino acids 183 to 201 of human α1-acid glycoprotein was synthesized and the peptide was conjugated to KLH via its terminal cysteine using a maleimide crosslinker and rabbits were immunized four times with the KLH-conjugated peptide. Anti-AGP antibodies were purified from sera using the synthetic peptide coupled to 2 mL of UltraLink Iodoacteyl resin (Pierce, Rockford, IL, USA).

### Purification of AGP

AGP was isolated from plasma samples using a two-step ion exchange chromatography method according to Asao et al. [18]. One mL plasma samples were applied to a HiTrap Desalting column (GE Healthcare, Uppsala, Sweden) equilibrated with 20 mM citrate-phosphate buffer, pH 4. The desalted peak was applied to a HiTrap DEAE column (GE Healthcare) equilibrated with 20 mM citrate-phosphate buffer, pH 4. Fractions containing AGP were eluted with 20 mM citrate-phosphate buffer, pH 7, containing 200 mM NaCl, pooled and applied on two joined HiTrap Desalting columns equilibrated with 20 mM citrate-phosphate buffer, pH 4. The desalted peak was applied to a HiTrap SP column (GE Healthcare) equilibrated with 20 mM citrate-phosphate buffer, pH 4, and AGP was eluted with 20 mM citrate-phosphate buffer, pH 4.8. The eluted fractions were dialyzed against water and lyophilized.

### Enrichment and detection of S2 and AAL-binding AGP from plasma samples

S2 and recombinant AAL (rAAL) were biotinylated using EZ-Link Sulfo NH-LC Biotinylation kit (Pierce, Rockford, IL, USA) according to the manufacturer’s protocol. A 5 molar fold excess of biotin reagent per protein sample was used for the biotinylation. The biotin/protein ratio was determined using a HABA/avidin-assay and calculated to 1 biotin moiety per S2 molecule and 2 biotin moieties per AAL molecule. EZview Red Streptavidin Affinity Gel (Sigma-Aldrich, Saint Louis, Mo, USA) was used for the enrichment of S2 and AAL-binding glycoproteins from plasma samples according to the manufacturer’s protocol. Briefly, 20 μL of EZview Red Streptavidin Affinity Gel, equilibrated with phosphate buffered saline (PBS) pH 7.4 (Medicago, Uppsala, Sweden), were mixed with 10 μg of S2 or AAL and incubated for one hour at room temperature. The lectin gel was washed once with PBS, once with 3% bovine serum albumin (BSA) (Sigma-Aldrich) in PBS and three times with PBS before 1 μL of plasma sample was added. After one hour of incubation the lectin-gel was washed three times with PBS and mixed with Laemmli sample buffer (Bio-Rad, Hercules, CA, USA). The enriched glycoproteins were separated under denaturing conditions on a gradient (4–20%) Mini-protean TGX gel (Bio-Rad) and transferred to a PVDF membrane for Western blot analysis. The membrane was blocked for 1 h with 3% BSA in PBS and incubated with rabbit anti-human-α1 acid glycoprotein 058 at 1 μg/mL in 3% BSA in PBS, followed by a goat anti-rabbit IgG HRP-conjugated antibody diluted 1:20 000 in 1% BSA in PBS. ECL substrate (GE Healthcare, Buckinghamshire, UK) was used for the detection of enriched AGP.

### Reverse lectin ELISA

Microtiter plates (Maxisorp, Nunc) were coated with S2 at 5 μg/mL in 0.05 M carbonate-bicarbonate buffer, pH 9.6 (Medicago), overnight at 4°C. Wells were blocked with 3% BSA in PBS (Medicago) for 1 h at room temperature (RT). Plates were washed 3 times with PBS + 0.05% Tween 20 (PBST) before the patient plasma samples, diluted 1:50 in PBS containing 1% BSA, were added and incubated shaking at 200 rpm for 1h at RT. After washing the wells as above the rabbit anti-human-α1 acid glycoprotein antibody 058 at 1 μg/mL in 1% BSA in PBS was added and incubated for 1 h with gentle shaking at RT. Bound anti-human-α1 acid glycoprotein antibodies were detected using a horseradish peroxidase conjugated goat anti-rabbit IgG (Jackson ImmunoResearch Laboratories, West Grove, PA) and O-phenylenediamine di-hydrochloride substrate (Sigma-Aldrich). The reaction was stopped after 30 minutes with addition of 25 μL/well of 1 M H_2_SO_4_ and the plate was read at 490 nm using a VERSAmax microplate reader (Molecular Devices Corporation).

### Traditional lectin ELISA

Microtiter plates (Maxisorp, Nunc) were coated with a rabbit polyclonal antibody directed towards human AGP (Sigma-Aldrich) at 10 μg/mL in 0.05 M carbonate-bicarbonate buffer, pH 9.6 (Medicago), overnight at 4°C. Wells were blocked with 3% BSA in PBS (Medicago) for 2 h at room temperature (RT). Plates were washed 3 times with PBS + 0.05% Tween 20 (PBST) before the patient plasma samples, diluted 1:50 in PBS containing 1% BSA, were added and incubated shaking at 200 rpm for 1h at RT. After washing the wells as above biotinylated S2, at a concentration of 2 μg/mL in 1% BSA-PBS, was added and incubated for 1 h shaking at 200 rpm at RT. Bound S2 was detected using an alkaline phosphatase conjugated ExtrAvidin (Sigma-Aldrich) and p-Nitrophenyl phosphate substrate (Sigma-Aldrich). The amount of bound S2 was measured at 405 nm using a VERSAmax microplate reader (Molecular Devices Corporation).

### Routine analysis

AFP concentrations were determined in the clinical routine lab at Linköping University Hospital on a COBAS e602 analyzer (Roche Diagnostics, Rotkreuz. Switzerland) and AGP concentrations were determined in the clinical routine lab at Kalmar County Hospital on a BN ProSpec system (Siemens, Erlangen, Germany).

### Statistical analysis

All statistical analyses were performed using IBM SPSS 23. Statistical differences between groups were determined using Tukey’s multiple comparison test in ANOVA. Binomial logistic regression was used to evaluate the combination of multiple markers. A *p* value of <0.05 was defined as being statistically significant. Receiver operating characteristics (ROC) curves and column scatter plots were generated with GraphPad Prism 5 (La Jolla, CA).

## Results

### Enrichment of AAL- and S2-positive AGP glycoforms from plasma samples

Recombinant full-length AAL was bound to agarose beads and used to capture fucosylated glycoforms in plasma samples from four patients with HCC, four patients with cirrhosis, two patients with chronic hepatitis and from a pool of normal plasma. The captured glycoproteins were analyzed by Western blot using antibody 058 directed against a non-glycosylated epitope of AGP.

**Fig 1:**
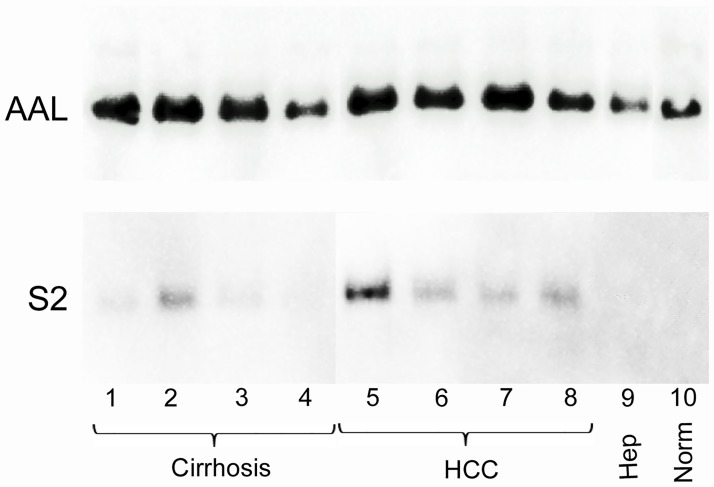
Western blot of AAL and S2 enriched α1-acid glycoprotein (AGP) from patient plasma samples. Lanes 1–4 represent cirrhosis samples, lanes 5–8 HCC samples, lane 9 a hepatitis sample and lane 10 a normal plasma sample. AGP was detected using the anti-human AGP antibody 058.

AGP was detected in all patient samples and in the normal control. However more AAL-bound AGP was detected in the samples from HCC patients and cirrhosis samples compared to the hepatitis samples and normal control. There was no visible difference between the amount of AAL-bound AGP between cirrhosis and HCC samples. (Fig 1, top panel).

When the single-site form of AAL, S2, comprising one of the 5 binding sites for fucose in native AAL, was used to enrich fucosylated plasma glycoproteins from the same samples, there was very little or no visible AGP staining in the normal sample and the hepatitis samples. Furthermore, in contrast to native AAL, S2 bound more AGP in the HCC samples compared to the cirrhosis samples. The cirrhosis samples showed no or moderate staining of S2-bound AGP whereas all samples from HCC patients showed staining of S2-bound AGP, indicating that S2 may be more specific towards HCC glycosylation (Fig 1, bottom panel).

### Development of a reverse S2 lectin ELISA

**Fig 2:**
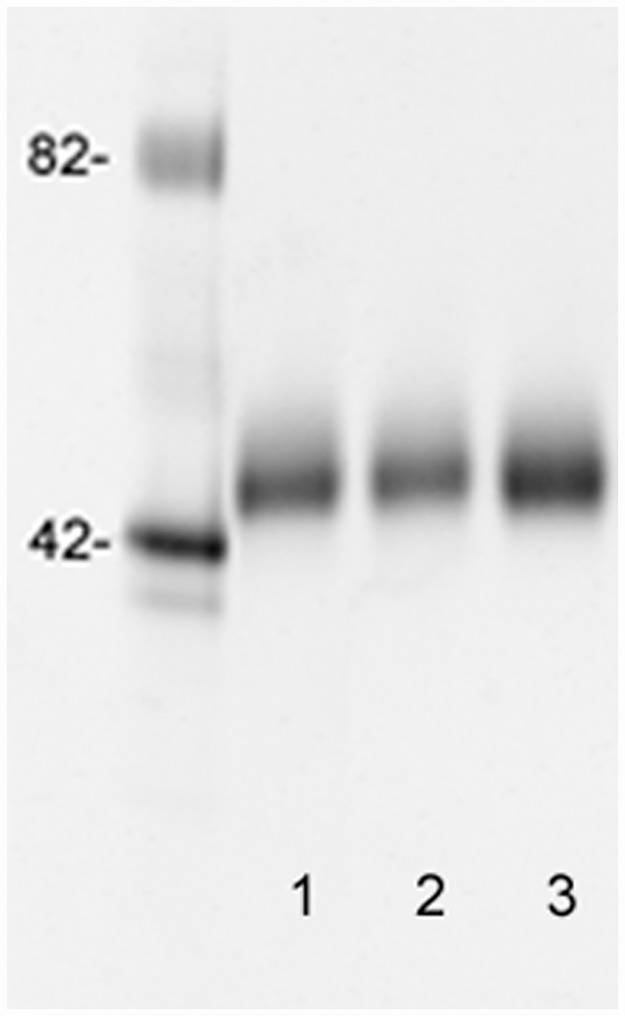
Pro-Q-Emerald 300 stained SDS-PAGE of purified AGP from a HCC plasma sample (lane 1), a cirrhosis plasma sample (lane 2) and a normal plasma sample. Molecular weight markers are shown to the left.

AGP was purified from one patient with HCC, one patient with cirrhosis and from the normal pool and used in a reverse lectin ELISA to measure the binding of AGP. The purified AGPs showed a single band at approximately 45-kDa when analyzed by SDS-PAGE (Fig 2). S2 was coated to microtiter wells and increasing concentrations of purified AGP samples was measured using the 058 antibody as described in “Materials and Methods”.

**Fig 3:**
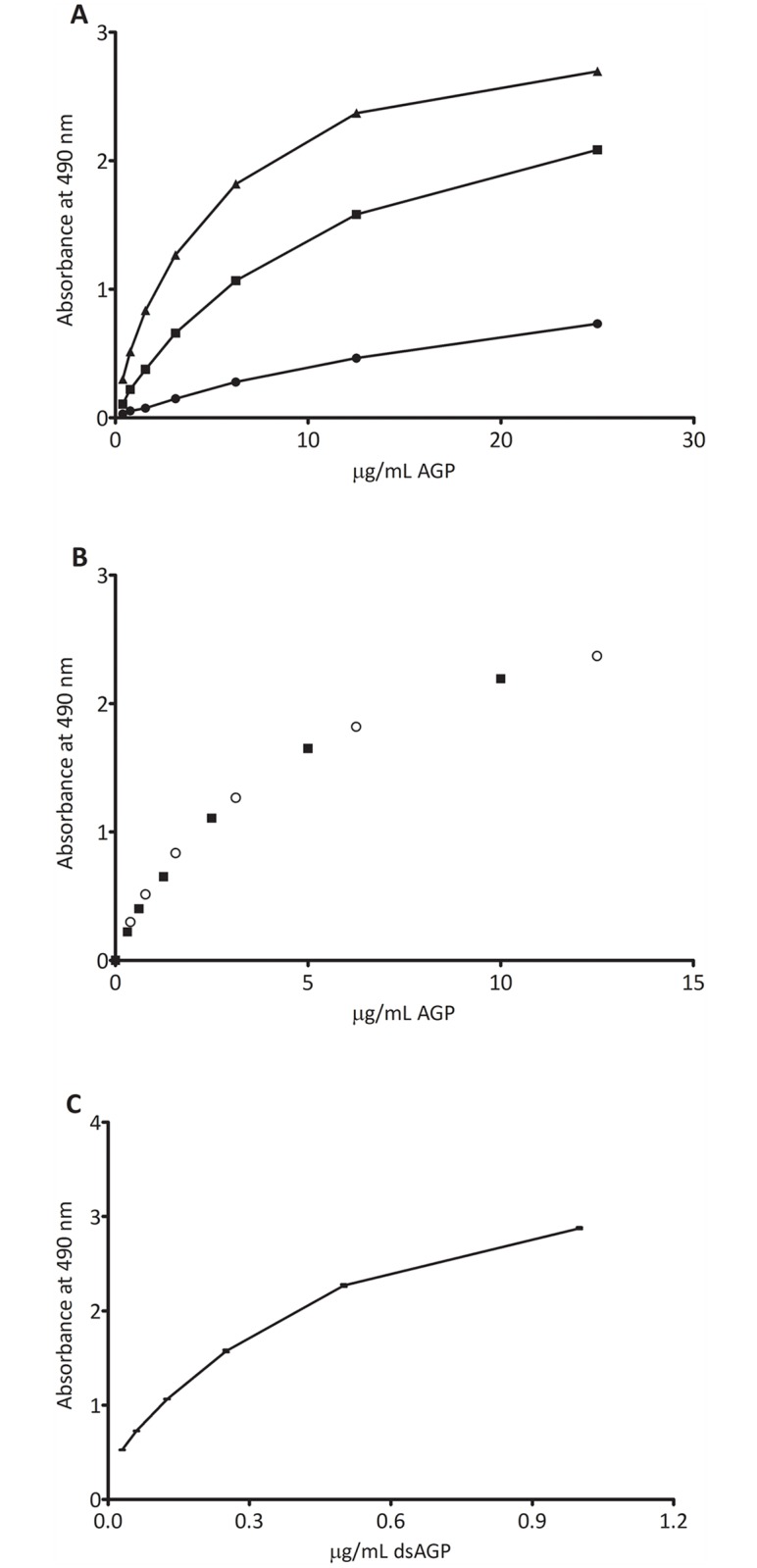
**A**. Binding of different concentrations of purified AGP from patient samples to S2 in the reverse S2 lectin ELISA. AGP purified from a patient with HCC (black triangles), AGP purified from a cirrhosis patient (black squares) and AGP purified from a normal sample (black circles). B. Comparison of binding of different concentrations of purified AGP from a HCC patient sample diluted in 1% BSA in PBS (circles) and diluted in normal serum (black squares). **C.** Binding of different concentrations of dsAGP to S2 in the reverse S2 lectin ELISA assay.

There was a dose-dependent increase in signal from all three samples. However AGP isolated from the normal pool showed very low absorbance values in the reverse ELISA consistent with the lectin precipitation data. The sample from the cirrhosis patient showed intermediate absorbance values whereas AGP purified from the HCC patient showed high absorbance values. This indicated that the reverse lectin ELISA specifically detect glycosylation differences between the different samples (Fig 3A).

### Effect of plasma

The reverse S2 lectin ELISA was further validated using a normal plasma sample with addition of purified HCC-AGP to assess matrix effects. Using a dilution of 1:50 of a normal plasma sample gave the same background signal as a negative control where plasma was omitted from the assay (OD = 0.2). This indicates that essentially no AGP from a normal plasma sample bound to S2. Addition of HCC-AGP to a normal plasma sample in increasing concentrations showed a dose dependent increase in absorbance which correlated with the increase in absorbance seen with samples without added plasma. This indicates that an increase in S2-bound AGP could be accurately measured in a plasma sample (Fig 3B).

### Optimizing coating concentration of S2 and antibody dilution

To optimize the coating concentration of S2 and the dilution of primary and secondary antibodies in the reverse lectin assay, absorbance values were measured for a HCC-plasma sample and a normal sample (background) using different coating and antibody concentrations. It was found that a coating concentration of 5 μg/ml S2, primary antibody concentration of 1 μg/mL and secondary antibody dilution of 1:40 000 gave an optimal signal to background ratio of over 10 (data not shown).

A standard curve was constructed using a desialylated form of AGP (dsAGP). Although dsAGP would not reflect the S2-bound form of AGP in the patient samples it could be used to relate the absorbance values obtained in the reverse lectin assay (Fig 3C).

### Reproducibility and precision of assay

Intra-assay precision of the assay was determined by repeated analysis of a dsAGP sample on the same plate (96 wells). The coefficient of variation (CV) for intra-assay variation was 1.2% (not shown).

Inter-assay variability was determined by analyzing two HCC samples with intermediate and high absorbance values on three different plates. The CV for HCC (intermediate) and for HCC (high) was 2.8% and 5.0% respectively (not shown).

### Determination of S2-bound AGP in patient plasma samples

**Fig 4:**
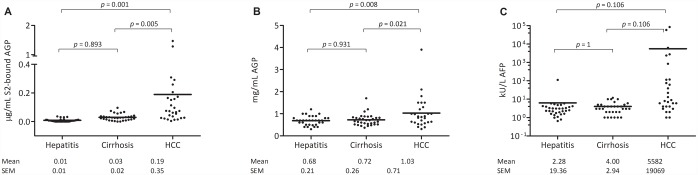
Scatter plots of levels of S2-bound AGP (as μg/ml of dsAGP standard) (A), AGP (B), and AFP (C) in patients with hepatitis, cirrhosis and HCC.

The reverse S2 lectin ELISA was used to determine the S2-bound AGP in patient plasma samples from hepatitis patients, cirrhosis patients and HCC patients. There was a significant increase in S2-bound AGP in plasma from HCC patients compared to plasma from both hepatitis patients (*p* = 0.001) and cirrhosis patients (*p* = 0.003). There was no significant increase in the level of S2-bound AGP when comparing cirrhosis patients to hepatitis patients (Fig 4A).

**Fig 5:**
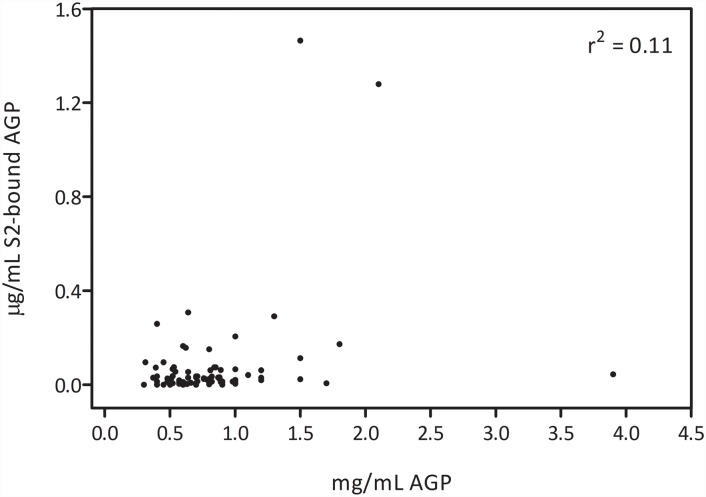
Correlation plot of levels of AGP and S2-bound AGP. The r square value is 0.11.

In the reverse ELISA set-up, changes in concentration of AGP in the patient samples could potentially influence the detected glycosylation changes. Therefore, concentration of AGP was measured in all samples. AGP concentrations showed variation from 0.3 to 3.9 mg/ml. There was a significant increase in AGP concentration between HCC and hepatitis samples (*p* = 0.008) and between HCC and cirrhosis samples (*p* = 0.02) but no significant differences in AGP concentration between LC and hepatitis patients (Fig 4B). However, there was no correlation between the S2 bound AGP signal and the AGP concentration (Fig 5) indicating that differences in AGP concentration did not affect the diagnostic performance of the reverse S2 lectin ELISA.

For comparison, the levels of AFP was also measured in the samples. The mean value of AFP was 5581 ng/ml in patients with HCC compared to 4 and 6 ng/ml in patients with cirrhosis and hepatitis, respectively. However these differences were not found to be significant (Fig 4C).

**Fig 6:**
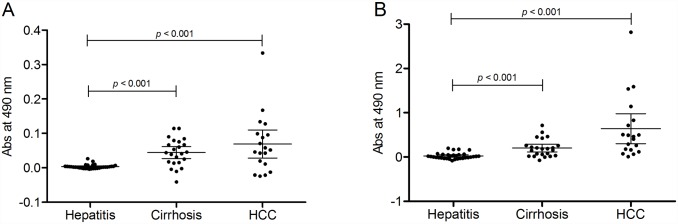
Scatter plots of bound fucosylated AGP in a traditional lectin ELISA (A) and in a reversed S2 lectin ELISA (B).

Hepatitis, cirrhosis and HCC patient samples were also analysed using a traditional lectin ELISA to compare the results from a reversed S2 lectin ELISA to the results from a traditional lectin ELISA. AGP in plasma samples was captured on anti-human AGP coated plates and detected with biotinylated S2. The absorbance signals, after subtraction of the background signal, were in the range of 0 to 0.2 using the traditional lectin ELISA (Fig 6A) compared to the 10 time higher range of 0 to 2 in the reversed lectin ELISA (Fig 6B). There was a corresponding elevation of HCC samples in each assay.

### Statistical analysis of S2-bound AGP

**Fig 7:**
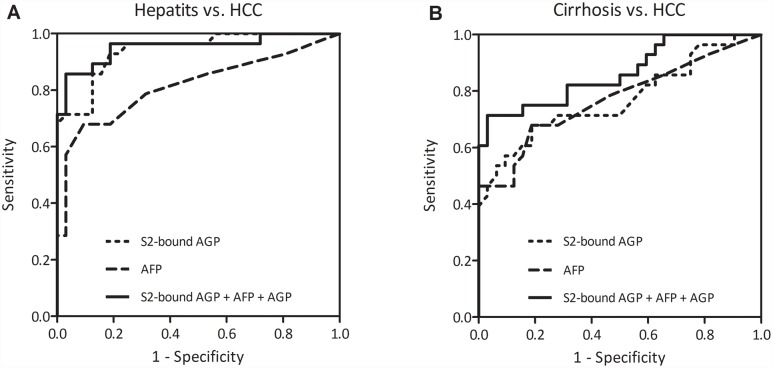
ROC-analyses of S2-bound AGP, AFP and a combination of S2-bound AGP, AFP and AGP to differentiate HCC from hepatitis (panel A) and HCC from cirrhosis (panel B).

Receiver operating characteristics (ROC) analysis was performed to determine the overall performance for each marker to differentiate between different patient populations. When differentiating HCC from hepatitis, the area under curve (AUC) for S2-bound AGP was 0.94. Differentiation of HCC from cirrhosis gave an AUC of 0.77 (Fig 7, Table 2). AFP had a similar performance as S2-bound AGP with an AUC of 0.77 for differentiation of HCC and cirrhosis and an AUC of 0.82 for differentiation of HCC and hepatitis (Fig 7, Table 2). ROC analysis of AGP concentration showed that AGP concentration by itself had poor performance in differentiating HCC from Hepatitis (AUC 0.66) and HCC from cirrhosis (AUC 0.65).

**Table 2:** ROC-analysis (HCC vs. hepatitis and cirrhosis).

Marker	Hepatitis vs. HCC	Cirrhosis vs. HCC
	AUC (95% CI)	AUC (95% CI)
AFP	0.815 (0.699–0.93)	0.77 (0.646–0.894)
AGP	0.664 (0.522–0.806)	0.653 (0.509–0.797)
S2-bound AGP	0.941 (0.886–0.997)	0.77 (0.644–0.893)
S2-bound AGP +AFP	0.942 (0.887–0.997)	0.826 (0.713–0.939)
S2-bound AGP + AGP	0.949 (0.893–1.000)	0.816 (0.706–0.926)
S2-bound AGP + AFP + AGP	0.952 (0.896–1.000)	0.864 (0.769–0.959)

AUC, area under the curve; CI, confidence interval

### Combinatorial analysis of S2-bound AGP, AFP and AGP in detection of HCC

Since there was an increase in the mean value of both S2-bound AGP, AFP and AGP when comparing HCC from cirrhosis patients, the performance of these markers were further analyzed using a combination of any two or all three markers using logistic regression analysis. A combination of all three markers showed the best differentiation between HCC and cirrhosis with an AUC of 0.86 and differentiation between HCC and hepatitis with an AUC of 0.95 (Fig 7, Table 2). The combination of S2-bound AGP with AFP and the combination of S2-bound AGP with AGP also showed improved performance than any of the markers used alone (Table 2).

## Discussion

Several studies have indicated an increase in fucosylation of plasma glycoproteins connected to development of both cirrhosis and HCC [19]. In studies where fucosylated proteins were captured by the fucose binding lectin AAL an increase of the amount of AAL-bound AGP in patients with HCC has been reported [20]. This suggested that AAL-bound AGP may be used as a marker for HCC development.

In the present study AAL-bead precipitation showed that there was an increase in AAL-bound AGP in both cirrhosis and HCC patient samples compared to normal. However, S2-bead precipitation showed that there was a tendency that HCC-patients had an increased level of S2-bound AGP in their plasma as compared to both cirrhosis patients and hepatitis patients.

S2, which is a single-site variant of AAL, has a more restricted specificity towards fucosylated oligosaccharides compared to the native form of AAL. In contrast to AAL, S2 will not bind sialylated/fucosylated epitopes such as SLe^x^ and SLe^a^ and has strongest affinity towards α1–6 fucosylated (core-fucosylated) and multifucosylated oligosaccharide structures [17]. Since S2 also binds to fucosylated structures with a lower overall affinity than AAL, it is possible that only multifucosylated glycoforms of AGP will have affinity enough to bind to S2. AGP express primarily highly branched complex-type N-linked oligosaccharides and previous studies have shown that development of HCC induces increased branching and fucosylation of AGP glycans with oligosaccharides expressing up to 3 fucose molecules per oligosaccharide chain [21, 22].

Based on the lectin agarose bead precipitation data a reverse lectin immunoassay was developed where S2 was coated to microtiter plates and the amount of lectin bound AGP was measured using an antibody directed against a non-glycosylated epitope of AGP. There are several abundant fucosylated plasma glycoproteins that potentially could bind to S2 and compete for binding of AGP. However, since S2 binds fucose with considerably lower affinity than a multivalent lectin such as AAL, it may be that only a small subset of glycoproteins carrying multiply fucosylated oligosaccharide structures will be captured efficiently by S2. The increased signal obtained when adding increasing amounts of HCC-AGP to serum samples indicates that the lectin coated surface is not saturated with other fucose-containing proteins which would substantiate this hypothesis.

Traditional lectin ELISA assays are commonly based on coating the microtiter plates with an antibody directed towards the target glycoprotein and then probing with lectins. These assays often suffer from low signal intensities and high background signals due to the presence of fucosylated carbohydrates on the capturing antibody. When the reversed S2 lectin ELISA was compared to the traditional lectin ELISA, using antibody capture and S2 detection, it was evident that signals obtained using the reverse S2 lectin ELISA were much more prominent. However, the weak signals obtained using the traditional lectin ELISA did also indicate an increased fucosylation in the HCC samples. Immunoglobulins such as IgG and IgM are known to carry fucosylated oligosaccharides and therefore there is often a need to pretreat the capturing antibodies with periodate oxidation to destroy fucose residues which could decrease antibody activity [23]. However, generally antibodies do not carry highly branched multiply fucosylated oligosaccharides, which may explain the weak binding to S2 and low background signals obtained in the reverse S2 lectin ELISA without the use of prior periodate oxidation.

The reverse lectin assay relies on that the target glycoprotein is present in relatively high proportion of the total glycoprotein content in the plasma sample [24]. It is important that the plasma sample can be diluted enough to avoid saturation of the capturing lectin with other fucosylated plasma glycoproteins. A reverse lectin ELISA based on AAL capture of fucosylated haptoglobin was recently used to differentiate patients with ovarian cancer from normal controls, showing the feasibility of this approach for other highly abundant glycoproteins such as haptoglobin [25]. To analyze matrix effects of other components in plasma, for example other fucosylated glycoproteins that potentially could bind to the S2 coated surface, we used purified AGP from a HCC patient. It was found that the signals obtained in the reverse S2 ELISA using HCC-AGP diluted in buffer corresponded to the signals obtained when HCC-AGP was diluted in plasma. This indicated that there were no matrix effects.

To compare samples analyzed on different microtiter plates, desialylated AGP was used to establish a standard curve. Normal AGP does not bind well to S2 and could therefore not be used as a standard. However, it was found that the desialylated form of AGP gave a good response in the reverse S2 lectin ELISA. Normally AGP primarily express fucose bound as terminal Sialyl-Le^x^ epitopes [26]. Whereas S2 show very low affinity towards this epitope, S2 will bind to the Le^x^ structures that will be exposed on AGP after desialylation. It cannot be excluded that desialylated structures also are partly present in HCC-AGP, however ELISA tests performed with *Ricinus Communis* lectin that is directed towards terminal galactose showed no increase in binding to HCC-AGP, indicating that this is not the case (data not shown). Although a standard curve based on desialylated AGP will not give an absolute value of the concentration of S2-bound AGP, it will enable standardization of the assay and comparisons of different analyses.

It was found that the reverse S2 lectin ELISA significantly discriminated between both cirrhosis and HCC samples compared to hepatitis samples. Furthermore the assay discriminated significantly between cirrhosis and HCC samples with an AUC value of 0.77, which was similar to what was found when using AFP. Importantly, it was found that AFP signals and S2-bound AGP signals were not completely overlapping. Thus, a combination of AFP and S2-bound AGP gave an AUC of 0.83 in differentiating HCC from cirrhosis. This indicated that the reverse S2 lectin ELISA could be a valuable complement to AFP for aid in the diagnosis of HCC. However, the analyses were performed on a limited amount of patient samples and further validation studies performed on larger cohorts are needed to establish this.

Concentration of AGP in the patient samples differed significantly between hepatitis, cirrhosis and HCC samples. This is consistent with a previous study showing elevated levels of AGP in HCC patients [27]. Importantly, there was no correlation between the AGP concentration and the level of S2- bound AGP. Thus, the observed changes in S2 bound AGP could not be explained by merely reflecting concentration differences in total serum AGP. This indicates that the reverse S2 ELISA measures the concentration of specific glycoforms of AGP that are not correlated to AGP concentration. A combination of S2-bound AGP, AFP and AGP further increased the performance in differentiating between HCC and cirrhosis patients giving an AUROC of 0.86.

Recent studies have indicated that the etiology of HCC is important for the fucosylation degree of plasma glycoproteins. It was found that bifucosylation of haptoglobin oligosaccharides correlated better with development of HCC in patients with an etiology of HBV-related disease than with development of HCC in patients with HCV-related or alcohol-related liver disease [21]. It would be interesting to analyze whether this also applies to S2-bound AGP.

In conclusion, the present study confirms that the measurement of increased fucosylation of plasma glycoproteins such as AGP can be valuable in differentiating HCC patients from patients with benign diseases, such as cirrhosis. Furthermore the study indicates that using carefully designed reagents in a reverse lectin assay format can increase the specificity towards multifucosylated glycoforms of specific plasma proteins and overcome some of the limitations that are often encountered using traditional lectin assays.
